# FKBP51, AmotL2 and IQGAP1 Involvement in Cilastatin Prevention of Cisplatin-Induced Tubular Nephrotoxicity in Rats

**DOI:** 10.3390/cells11091585

**Published:** 2022-05-09

**Authors:** Rebeca González-Fernández, María Ángeles González-Nicolás, Manuel Morales, Julio Ávila, Alberto Lázaro, Pablo Martín-Vasallo

**Affiliations:** 1 Laboratorio de Biología del Desarrollo, UD de Bioquímica y Biología Molecular and Centro de, Investigaciones Biomédicas de Canarias (CIBICAN), Universidad de La Laguna, Av. Astrofísico Sánchez s/n., 38206 La Laguna, Spain; refernan@ull.edu.es (R.G.-F.); javila@ull.edu.es (J.Á.); 2 Renal Physiopathology Laboratory, Department of Nephrology, Instituto de Investigación Sanitaria Gregorio Marañón, Hospital General Universitario Gregorio Marañón, 28007 Madrid, Spain; rengac@yahoo.es; 3 Department of Medical Oncology, Nuestra Señora de Candelaria University Hospital, 38010 Santa Cruz de Tenerife, Spain; mmoraleg@ull.edu.es; 4 Department of Physiology, School of Medicine, Universidad Complutense de Madrid, 28040 Madrid, Spain

**Keywords:** FKBP51, IQGAP1, AmotL2, cisplatin toxicity

## Abstract

The immunophilin FKBP51, the angiomotin AmotL2, and the scaffoldin IQGAP1 are overexpressed in many types of cancer, with the highest increase in leucocytes from patients undergoing oxaliplatin chemotherapy. Inflammation is involved in the pathogenesis of nephrotoxicity induced by platinum analogs. Cilastatin prevents renal damage caused by cisplatin. This functional and confocal microscopy study shows the renal focal-segmental expression of TNFα after cisplatin administration in rats, predominantly of tubular localization and mostly prevented by co-administration of cilastatin. FKBP51, AmotL2 and IQGAP1 protein expression increases slightly with cilastatin administration and to a much higher extent with cisplatin, in a cellular- and subcellular-specific manner. Kidney tubule cells expressing FKBP51 show either very low or no expression of TNFα, while cells expressing TNFα have low levels of FKBP51. AmotL2 and TNFα seem to colocalize and their expression is increased in tubular cells. IQGAP1 fluorescence increases with cilastatin, cisplatin and joint cilastatin-cisplatin treatment, and does not correlate with TNFα expression or localization. These data suggest a role for FKBP51, AmotL2 and IQGAP1 in cisplatin toxicity in kidney tubules and in the protective effect of cilastatin through inhibition of dehydropeptidase-I.

## 1. Introduction

Cisplatin is one of the most potent antineoplastics used in the treatment of various types of cancer; however, approximately one-third of patients undergoing cisplatin treatment experience acute kidney injury, with decreased glomerular filtration rate (GFR), increased serum creatinine and blood urea nitrogen (BUN), and electrolyte imbalance [1,2,3,4]. This cisplatin-induced nephrotoxicity limits the clinical use of the drug [1,4,5]. A wide body of experimental evidence indicates that cilastatin, a specific inhibitor of renal dehydropeptidase-I (DHP-I), can protect proximal tubular epithelial cells from the damage caused by cisplatin, without compromising the drug’s therapeutic effect on cancer cells [1,2,3,4,5,6,7,8].

This study aims to shed light on the mechanisms by which cilastatin protects kidney cells from cisplatin-induced toxicity. In particular, we looked at three proteins whose expression is significantly altered in leucocytes after platinum-derivative chemotherapy, namely [7] FKBP51 (FK506, tacrolimus-binding protein 51), AmotL2 (angiomotin-like 2) and IQmotif-containing GTPase-activating protein 1 (IQGAP1); these three proteins are involved in the regulation of proteins acting in a variety of processes, including cell growth, tumorigenesis, inflammation, immunity, cell plasticity and differentiation, and many others [9,10,11]. String analysis [12] and text mining show predicted and experimentally demonstrated interactions between FKBP51, AmotL2, and IQGAP1 [13].

FKBP51 is an immunophilin with peptidyl-prolyl cis-trans isomerase (rotamase) activity which acts as a co-chaperone associated with heat shock protein 90 (Hsp90), heat shock protein 70 (Hsp70) and p23; it plays a role in steroid receptor signaling, translocating from the cytosol to either the mitochondria or nucleus, where it modulates pathways involving protein kinase B (Akt), protein kinase A (PKA), nuclear factor-kappa B (NF-κB), transforming growth factor (TGF) and tumor necrosis factor alpha (TNFα) [14,15,16,17,18,19,20]. FKBP51 is abundantly expressed in tumours [21,22] and is involved in antiapoptotic processes in cancer cells [23]. Translocation of FKBP51 to the nucleus appears to be related to decreased cyclin D expression in the cell cycle [21]; also found in the nucleus is FKBP51s, an alternatively spliced isoform of FKBP51, which is induced by the co-inhibitory immune checkpoint PD-L1/PD1 [23].

AmotL2 is an angiostatin binding protein and member of the motin family [15]; it promotes vascular tube formation by regulating cell-cell interactions and also plays a role in maintaining apical-basal polarity. AmotL2 modulates signaling pathways such as the Hsp70-Bag3 pathway, in which it scaffolds the LATS1 and YAP proteins of the Hippo pathway [24]; it has also been shown to be involved in the epithelial to mesenchymal transition of a number of cancers [15].

IQGAP1 is an extraordinarily versatile scaffold protein that regulates numerous cellular processes and signaling pathways; it plays a role in complex cell functions such as tight junction formation, tissue and organ physiology, the cell cycle, angiogenesis, cell migration, cytoskeletal organization, and many others [25,26]. In the kidney, it regulates intercellular junctions that mediate glomerular filtration [25,27] and is involved in cytoskeleton formation and tubulogenesis [28,29].

For further information regarding protein structure, functional domains and other properties, see references [16] for FKBP51, [30] for Amotl2 and [31,32] for IQGAP1.

The aims of this study were to determine the precise localization of cisplatin-induced inflammation in the kidney, to elucidate whether FKBP51, Amotl2 and IQGAP1 play a role in this inflammation, and to determine the effects of cilastatin on the expression of these proteins and their cellular and subcellular localization.

## 2. Materials and Methods

### 2.1. Animals

Wistar Han (WKY, Rattus norvegicus) 7-week-old male rats, 250–270 g weight (Charles River Laboratories, Barcelona, Spain), were housed under controlled light (12 h light/dark cycle), temperature (23 ± 1 °C), and humidity (60 ± 10%), with free access to food consisting of Standarddiets, Altromin^®^ (Altromin Spezialfutter GmbH & Co, Lage, Germany) and tap water. The animals’ weight was checked before the experiment and just before slaughter. The study was approved by the Institutional Board for Animal Experiments of the Gregorio Marañón Hospital (registration code 07-2008) and animals were handled at all times according to legal regulations stipulated by RD 118/2021, of February 23, on the protection of animals used for experimental scientific purposes.

### 2.2. Drugs

Crystalline cilastatin was obtained from Merck Sharp and Dohme S.A. (Madrid, Spain) and cisplatin from Pharmacia (Barcelona, Spain). The vehicle for both drugs was normal 0.9% saline.

### 2.3. Experimental Protocols

Experiments were performed using 24 animals, randomized into 4 groups: untreated control rats (*n* = 6); cilastatin-treated rats (*n* = 6); cisplatin-treated rats (*n* = 6); and cilastatin-protected cisplatin-treated rats (*n* = 6). The animals were treated as follows:

- Cilastatin protected cisplatin-treated rats: Cisplatin was administered by intraperitoneal (IP) injection, as a single dose of 5 mg/kg body weight (bw). Cilastatin was administered IP at 75 mg/kg bw just before the time of cisplatin administration, and then every 12 h until the time of slaughter.
- Cisplatin-treated rats: Cisplatin was administered IP as a single dose of 5 mg/kg bw. Saline was administered IP in the same volume as cilastatin-treated groups just before the time of cisplatin administration, and then every 12 h until the time of slaughter.
- Cilastatin-treated rats: Saline was administered IP in the same volume as the cisplatin-treated groups. Cilastatin was administered IP at 75 mg/kg bw just before the time of saline administration, and then every 12 h until the time of slaughter.
- Control rats: saline was administered IP in the same volumes and regimens as in the cilastatin- and/or cisplatin-treated groups.

The cisplatin or saline injections were given in the same manner and volume (1 mL/100 g). The first dose of cilastatin was given just before the cisplatin injection. Saline (0.25 mL/100 g) was administered in place of cilastatin in the other groups. The dose and administration period of cisplatin was selected based on the proven effectiveness of the drug in inducing nephrotoxicity [1,33,34,35]. The dose of cilastatin was based on previous experience [1,36], which had shown that imipenem/cilastatin reduced Cyclosporin A (CsA)-induced nephrotoxicity. The urine from each animal was collected over the 24 h period before slaughter and the volume was measured. Five days after the first injection, all animals were anaesthetized with ketamine (10 mg/kg) and diazepam (4 mg/kg) and killed by exsanguination. Total blood samples were collected by insertion of a cannula into the abdominal aorta, and serum was separated for biochemical analysis. Kidneys were perfused with cold saline and quickly removed. Kidney samples were fixed in 4% paraformaldehyde (24 h) and paraffin-embedded.

### 2.4. Renal Function Monitoring

Serum and urine creatinine concentrations and Na^+^ and BUN levels were measured automatically using the Dimension RxL autoanalyzer (Dade-Behring, Siemens, Eschborn, Germany) in accordance wth the manufacturer’s recommendations. The GFR was estimated based on the creatinine clearance rate. Fractional excretion of sodium was calculated as: EFNa^+^ = ([Na + urine]/[Na^+^ serum]) × ([Creatinine plasma]/[Creatinine urine]) × 100. The fractional excretion of water was calculated using: EFH_2_O = (urine volume/glomerular filtration rate) × 100.

### 2.5. Renal Histopathological Studies

For light microscopy, paraffin-embedded cortex and medulla renal sections (4 µm thick) were stained with hematoxylin-eosin (H-E) (Sigma-Aldrich, St Louis, MO, USA). The kidney injury score was calculated using a previously described semiquantitative index [37]. Briefly, tubular damage scoring was defined as the blebbing of apical membranes, tubular cast formation, epithelial necrosis, tubular vacuolization and the presence of mitotic nuclei. Morphometric examination and scoring were performed by observers blinded to the animals’ treatment condition, using the following semiquantitative index: 0 points: no damage; 1 point: damage from 0 to 25% of the sample; 2 points: damage from 25 to 50% of the sample; 3 points: damage from 50 to 75% of the sample; 4 points: damage higher than 75% of the sample. The injury score was calculated as the sum of this semiquantitative assessment of tubular injury. Samples were examined with an Olympus BX-50 microscope (Olympus, Tokyo, Japan).

### 2.6. Western Blot Analysis

Western blotting was performed as previously described [36]. In brief, renal cortex protein extracts (50 µg) were assayed by electrophoresis and separated on commercial Precast TGX gels (Bio-Rad, Hercules, CA, USA) between 10% and 15% under reducing conditions. Subsequently, they were transferred by Transfer Pack, Midi Format, 0.2 μm nitrocellulose (Bio-Rad) in transfer buffer [Tris 48 mM pH 8.8, 39 mM glycine, 0.037% SDS and 20% methanol (*vol*/*vol*)] using the Trans-Blot^®^ Turbo^TM^ Transfer System (Bio-Rad). The membranes were blocked with 4% skim milk powder in phosphate-buffered saline (PBS)-Tween 20 0.1% for 1 h and incubated overnight at 4 °C with mouse anti-human RelA/NFΚB p65 monoclonal antibody, 1:500 (112A1021 Novus Biologicals, Bio-Techne R&D Systems, S.L.U., Madrid, Spain). As an internal control of the technique to verify equal protein loading, the membranes were also incubated with a mouse anti β-actin monoclonal antibody, 1:60,000 (Sigma Aldrich, Merck Life Science S.L.U., Madrid, Spain). The binding of the antibodies to both proteins was detected by peroxidase-conjugated anti-mouse IgG at 1:3000 (GE Healthcare, Buckinghamshire, UK) and identified by chemiluminescence with the Amersham™ ECL™ Prime Kit (GE Healthcare), using the Alliance 4.7 developer (Uvitec, Cambridge, UK).

Signal quantifications were carried out with the image analysis program Image J (Image Processing and Analysis in Java). The results were expressed in arbitrary densitometry units.

### 2.7. Immunohistochemistry

Four microns thick, 4% paraformaldehyde-fixed paraffin-embedded tissue sections were deparaffinized in xylene and hydrated in a graded series of alcohol baths. Heat-induced epitope retrieval was achieved by heating samples in sodium citrate buffer (pH 6.0) at 120 °C for 10 min in an autoclave. Non-specific sites were blocked with 5% bovine serum albumin in Tris-buffered saline (TBS) for 1 h at room temperature, then double immunofluorescence simultaneous staining was performed. Tissue sections were incubated overnight at 4 °C with mouse monoclonal anti-TNFα (52B83, dilution 1:150; #sc- 52746 Santa Cruz Biotechnology Inc., Dallas, TX, USA) and either rabbit polyclonal anti-AmotL2 (dilution 1:50; #LS-C178611; LifeSpan BioSciences, Seattle, WA, USA), rabbit polyclonal anti-FKBP51 (dilution 1:50; #ab46002; Abcam, Cam-bridge, UK) or rabbit polyclonal anti-human-IQGAP-1 (dilution 1:250; #ABT186 EMD; Millipore, Billerica, MA, USA). Samples incubated without primary antibodies were used as a negative control. Slides were incubated for 1 h at room temperature in the dark with a mixture of two secondary antibodies raised in different species and conjugated to different fluorochromes: fluorescein isothiocyanate (FITC)-conjugated goat polyclonal antibody against rabbit IgG (dilution 1:200; #F9887; Sigma-Aldrich, Saint Louis, MO, USA) and goat polyclonal antibody against mouse IgG DyLight 650 (dilution 1:100; #ab97018; Abcam). Slides were mounted with ProLong^®^Diamond Anti-fade Mountant with DAPI (Molecular Probes by Life technologies) to visualize cell nuclei.

Slides were analyzed using a Leica SP8 confocal microscope (Leica Microsystems, Wetzlar, Germany).

### 2.8. Image Analysis and Statistics

Three independent observers evaluated the specimens blindly. For H-E samples, observers evaluated 5 samples per rat and 15 to 20 fields per sample at 20× magnification. For confocal microscopy images (40× magnification), staining intensities were graded as absent (–), faint (+), moderate (++), or strong (+++). These cut-offs were established by consensus between investigators following an initial survey of all blindly coded sections. In cases where scoring data differed by more than one unit, the observers re-evaluated the sections to reach a consensus. In other cases, means were calculated.

Observers evaluated between 20 and 50 fields per sample. All images were captured at the same magnification (40×) and with the same levels of contrast and brightness, scoring them as absent (–), faint (+), moderate (++), or strong (+++).

Statistical analysis was carried out using SPSS (version 25 for Windows; IBM Corp., Armonk, NY, USA). For the analysis, – was quantified as 0, + as 1, ++ as 2, and +++ as 3. A dependence test (chi-square) was performed between the staining levels of each group of cells, and non-parametric techniques (median test and Kruskall–Wallis test) were used to analyze significant differences in the distribution of staining levels with respect to cell type.

Statistics for quantitative variables in Table 1, H-E histology analysis and western blot experiments were organized as mean ± standard error of the mean (SEM). Analysis of variance (ANOVA) was performed for normally distributed continuous variables, with the least significant difference test as a post hoc approach to determine specific group differences. Statistically significant differences were accepted for bilateral α values when *p* < 0.05.

## 3. Results

### 3.1. Cilastatin Improves Cisplatin-Induced Nephrotoxicity

Table 1 shows the effects of cisplatin and cilastatin administration on biochemical indicators of renal function. As expected, cisplatin-induced renal damage, characterized by increased serum creatinine, BUN levels, EFNa + and EFH_2_O, and decreased GFR compared with the control group. Cilastatin treatment partially or totally reversed these effects. Cisplatin caused polyuria in the animals, and although cilastatin appeared to decrease urine volume, this change was not statistically significant (Table 1). Administration of cilastatin alone had no effect on the parameters studied.

### 3.2. Cilastatin Prevents Histopathological Damage Induced by Cisplatin Administration

**Figure 1:**
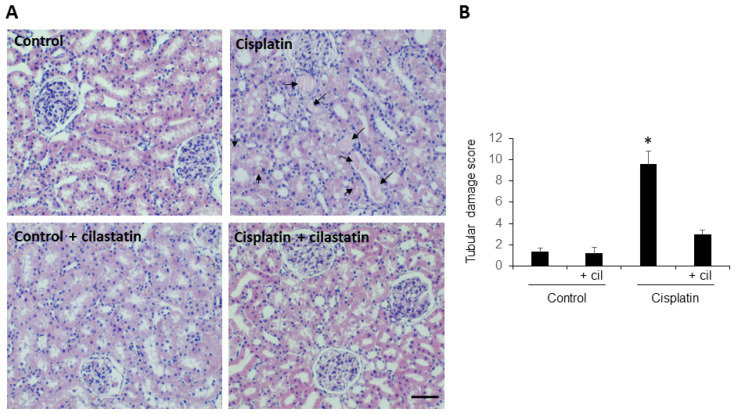
Cilastatin reduces histopathological damage induced by cisplatin administration. (**A**) Representative images of the hematoxilyn-eosin-stained renal cortex and medulla of control, cilastatin, cisplatin and cisplatin + cilastatin rats (20× magnification). Arrows point to intratubular protein casts from animals treated with cisplatin. The rest of the groups presented an apparently normal renal morphology. Bar = 100 µm. (**B**) Semiquantitative tubular damage score. Results are expressed as mean ± s.e.m., *n* = 6 animals per group. * *p* < 0.005 vs. all other groups.

H-E stained kidney sections from rats undergoing cisplatin treatment showed intratubular protein casts (a marker of damage) in 30 to 50% of tubules, depending on the field, with *p* < 0.005 compared to control (Figure 1, panel A). Samples from cilastatin-treated and cilastatin-protected cisplatin-treated rats presented an apparently normal renal morphology pattern. Figure 1, panel B shows the semiquantitative tubular damage score.

### 3.3. Cilastatin Diminishes Cisplatin-Induced Inflammatory NF-κB Upregulation

**Figure 2:**
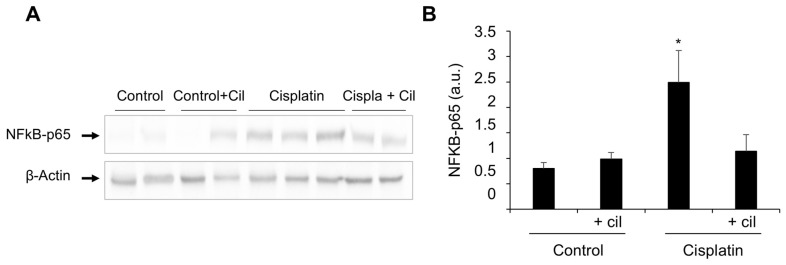
Cilastatin diminishes cisplatin-induced inflammatory NF-κB upregulation. (**A**) Representative photomicrograph of western blots of the p65 subunit of NF-κB in renal cortex. (**B**) Densitometric analysis of the p65 subunit on western blots. Cilastatin reduced the NF-κB values previously increased by cisplatin treatment. Results are expressed as mean ± SEM, * *p* < 0.03 vs. all other groups.

Western blots of protein extracts from the rats’ renal cortex showed a 20 to 25% increase in expression of the p65 subunit of NF-κB after cilastatin treatment (75 mg/kg bw IP every twelve hours), and a 3 to 4-fold increase after cisplatin treatment (5 mg/kg bw IP in a single dose). Coadministration of cilastatin with cisplatin diminished cisplatin-induced inflammatory NF-κB upregulation by 51–64%. A representative western blot of the p65 subunit of NF-κB in renal cortex is shown in Figure 2, panel A. Panel B shows densitometric analysis of the p65 subunit western blots (*p* < 0.03 vs. all groups).

### 3.4. Focal and Segmental Localization of TNFα Expression Elicited by Cisplatin in Rat Kidney Tubules

**Figure 3:**
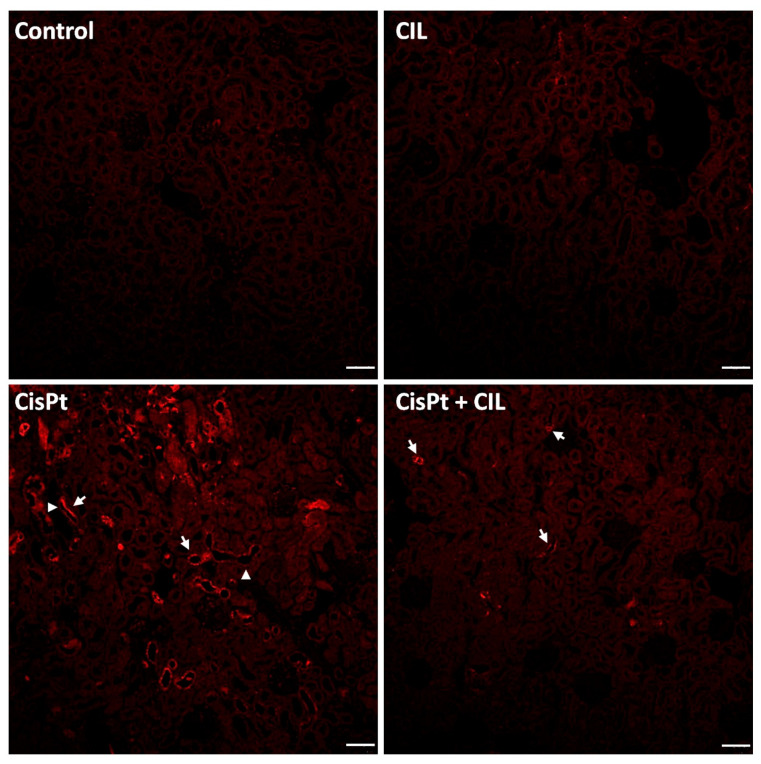
Focal and segmental localization of TNFα expression elicited by cisplatin in rat kidney tubules. Renal tubules of control and cilastatin (CIL)-treated groups show homogeneous basal expression of TNFα. In the cisplatin-treated group (CisPt), immunostaining for TNFα in tubules is heterogeneous, with positivity observed only in some tubules foci (white arrows), and sometimes confined to a segment of the tubule (arrowheads). Cilastatin-protected cisplatin-treated rat kidneys (CisPt-CIL), show scarce immunolocalization signals for TNFα (white arrows). 10× magnification. Bar = 40 µm.

Renal tubules of control and cilastatin-treated rats showed homogeneous basal expression of TNFα, with a few isolated tubular cells (<1/1000) showing higher TNFα-specific fluorescence. Immunostaining for TNFα in tubules of the cisplatin (CisPt) treated group was heterogeneous, only present in some tubules grouped in foci (white arrows). These made up 5–10% of the total sectioned tubules per field. Some TNFα+ tubules showed immunofluorescence only in a region/segment of the tubule (arrowheads). TNFα-negative cells within TNFα+ tubules range from one cell to 5 or 6. Few immunolocalization signals were seen for TNFα in cilastatin-protected cisplatin-treated rat kidneys, 5–6 fold higher than control samples (Figure 3, CisPt-CIL panel, white arrows).

### 3.5. Expression of FKBP51 in Cilastatin-Protected Cisplatin-Treated Rat Kidney

**Figure 4:**
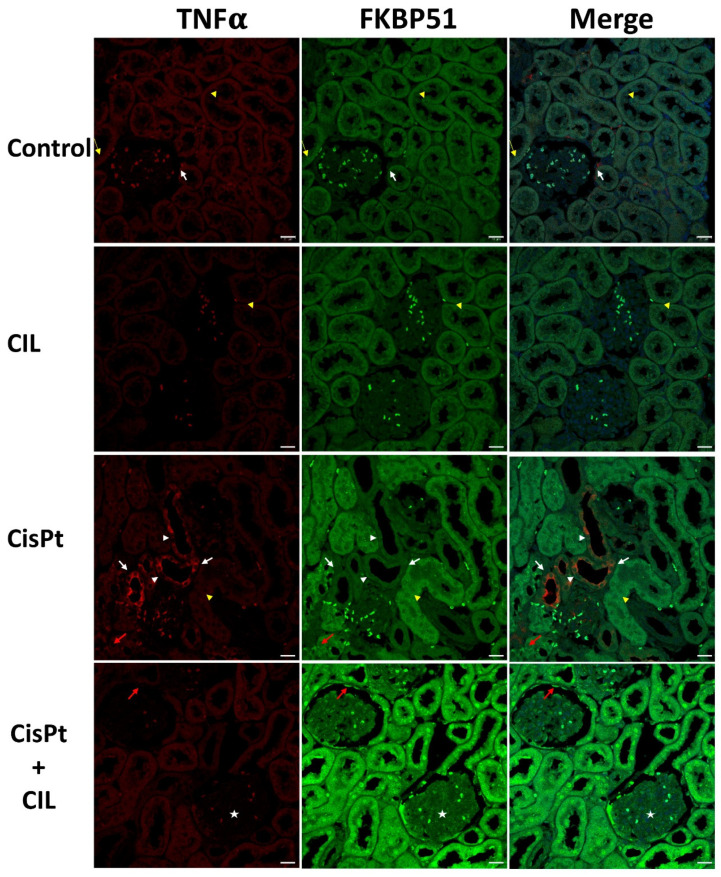
Expression of FKBP51 in cisplatin-treated and cilastatin-protected rat kidney. Control panels: tubular cells show low immunostaining of TNFα and FKBP51 in cytosol. White arrows point to small areas of higher TNFα fluorescence in the outer glomerular capsule cells, yellow thin arrows point to more intense FKBP51 fluorescence around nuclei in some tubule cells and yellow arrowheads point to some physiological cell blebbing. CIL panels: kidney tubule cells of cilastatin-treated rats show TNFα and FKBP51 immunostaining very similar to controls in localization and intensity. Yellow arrowheads point to some physiological cell blebbing. CisPt panels: kidney tubule cells of cisplatin-treated rats: TNFα-specific immunofluorescence appears heterogeneous within the same tubules, with several cells showing high levels in the cytosol (white arrows), while in some other cells the cytosolic signal is fainter (white arrowheads). Most tubules show a low-level signal, though slightly brighter than controls. Some tubules, such as the one indicated by the yellow arrowhead, present abundant cell blebbing. FKBP51-specific immunofluorescence presents two intensity levels: a low level in low TNFα-signal tubules and a higher level in the high TNFα-signal tubules. The immunosignal in nuclei has a multi-dot fluorescence pattern (red arrow). CisPt + CIL panels: kidney tubule cells of cilastatin-protected cisplatin-treated rats present a homogeneous fluorescence pattern for both TNFα and FKBP51. TNFα is cytoplasmic and at a similar level to controls, while FKBP51-specific fluorescence is of higher intensity in both nuclei and cytosol, being more prominent in nuclei (white arrow). TNFα fluorescence is very low in kidney glomeruli from all experimental conditions, while FKBP51 signal is low in control, CIL and CisPt groups, but has a clear signal in glomeruli cells (star) and capsule cells (red arrow). Bar = 20 µm.

Tubular cells of normal rat kidney (Figure 4, Control panel) showed basal low-intensity immunostaining levels for TNFα and FKBP51 in cytosol. Small areas presented higher TNFα immunofluorescence in the outer glomerular capsule cells (white arrows). Some tubule cells presented more intense FKBP51 perinuclear fluorescence (yellow arrows, where yellow arrowheads point to incidental physiological cell blebbing).

Cilastatin-treated rat kidney tubule cells showed immunostaining for TNFα and FKBP51 very similar in localization and intensity to that of controls. Yellow arrowheads in the CIL panels in Figure 4 point to some cell blebbing at the same levels of incidence as in the control group.

The CisPt panels of Figure 4 show kidney tubule cells of cisplatin-treated rats with a high level of TNFα-specific immunofluorescence in some tubules (foci), in the cytosol of most tubule cells (white arrows) and at a lesser intensity in the cytosol of some other cells within the same tubules (arrowheads). Most tubules showed a low-level signal, though slightly brighter than controls, and some TNFα+ tubules presented abundant cellular blebbing (arrowhead). See also Figure 6, CisPt panel, where cell blebbing is more prominent and abundant within the same field. FKBP51-specific immunofluorescence presented two well-differentiated intensity levels: a low level in low TNFα-signal tubules and a higher intensity in the high TNFα-signal tubules. Nuclei showed a multi-dot fluorescence pattern (red arrow).

**Figure 5:**
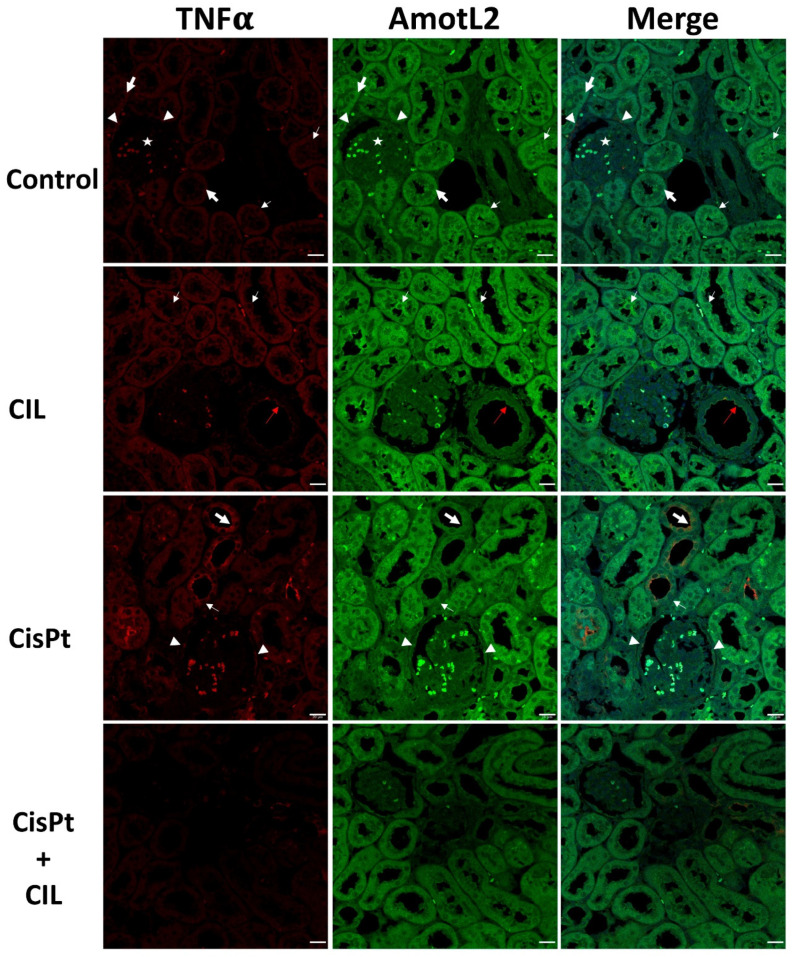
Expression of AmotL2 in the cisplatin-treated and cilastatin protected rat kidney. Control panels: tubular cells show low TNFα and homogeneous medium-intensity AmotL2 in cytosol (bold white arrows), most prominent in some cells around nuclei (thin white arrows). Some cells of the outer glomerular capsule show high staining intensity for TNFα and AmotL2 (white arrowheads). Glomerular cells show no TNFα signal and low AmotL2 staining (star). CIL panels: tubular cells of cilastatin-treated rats show homogeneous TNFα and AmotL2, with similar localization to controls, but at a slightly but definitely increased intensity level. AmotL2 staining is more intense around the nuclei of some cells, and most nuclei show AmotL2 staining in a dot-like pattern. Red arrows point to TNFα and AmotL2 immunostaining in the luminal side of endothelial cells of medium-sized vessels. CisPt panels: bold white arrows point to high-intensity TNFα-specific immunostaining in some tubule cells of cisplatin-treated rats; thin white arrows point to other cells within the same tubules with a lower staining intensity. In both cases intensity is higher at the apical brush border than in the cytosol. AmotL2/TNFα positive cells can be seen in the outer glomerular capsule (white arrowheads). AmotL2 fluorescence is lower in highly TNFα-positive cells. CisPt + CIL panels: kidney tubule cells of cilastatin-protected cisplatin-treated rats show a staining pattern very similar to that of controls, although both TNFα and AmotL2 signals are at a slightly lower intensity than in controls. 40× magnification. Bar = 20 µm.

**Figure 6:**
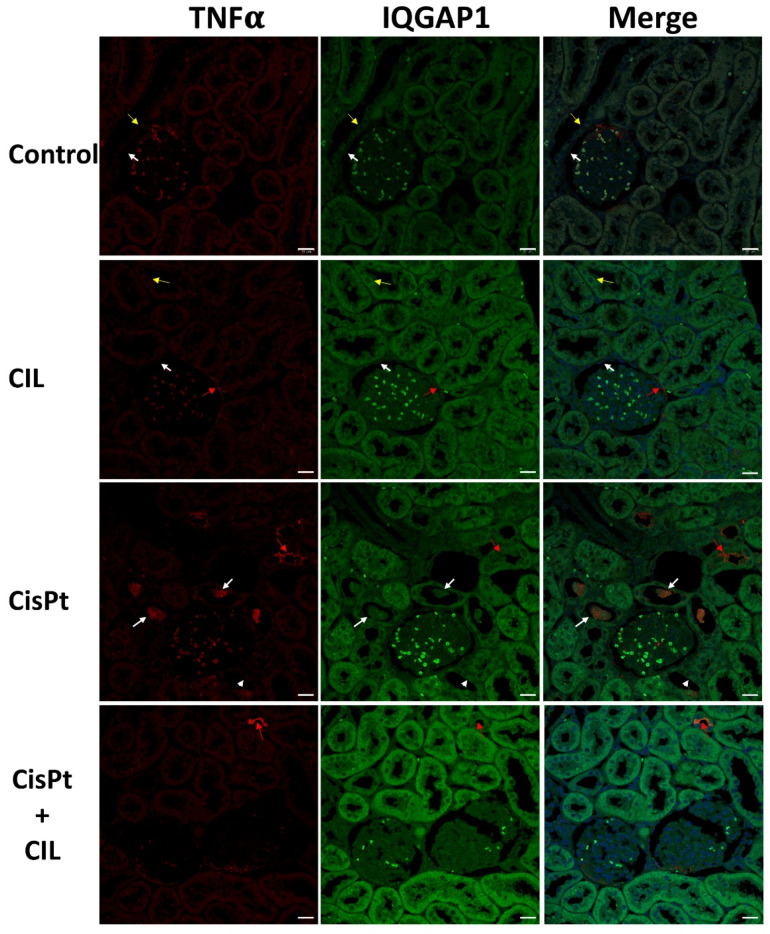
Expression of IQGAP1 in cisplatin-treated and cilastatin protected rat kidney. Control panels: low-intensity immunostaining for TNFα and IQGAP1 in cytosol of tubular cells. White arrows point to small areas of higher IQGAP1 immunofluorescence in outer glomerular capsule cells. Thin yellow arrows point to more intense IQGAP1 fluorescence around nuclei in some tubule cells. Glomerular cells show low expression of IQGAP1. CIL panels: kidney tubule cells of cilastatin-treated rats show slightly higher intensity TNFα and IQGAP1 staining compared to controls, with very similar localization. Red arrows indicate TNFα fluorescence in outer glomerular capsule cells. CisPt panels: kidney tubule cells of cisplatin-treated rats. High intensity TNFα fluorescence is found in the cytosol of some tubule cells inside the tubular light, as pluricellular blebbing bodies (white arrows). Yellow arrows indicate TNFα fluorescence at the luminal-apical border of some tubular cells and, at a much lower intensity (similar to controls) in the cytosol of other cells (arrowheads). CisPt + CIL panels: kidney tubule cells of cilastatin-protected cisplatin-treated rats have homogeneous TNFα and IQGAP1 fluorescence. TNFα is cytoplasmic and at a similar level to controls. A few tubular cells show TNFα fluorescence (yellow arrow). IQGAP1 fluorescence is of higher intensity. TNFα and IQGAP1 fluorescence was of very low intensity in kidney glomeruli from all experimental conditions. 40× magnification. Bar = 20 µm.

Kidney tubule cells of cilastatin-protected cisplatin-treated rats (CisPt + CIL) had a homogeneous fluorescence pattern for both TNFα and FKBP51. TNFα was located in cytoplasm and at a very similar level to control rats, while FKBP51 was higher in both nuclei and cytosol, being more prominent in nuclei (white arrows in Figure 4, CisPt + CIL panels). In general, low TNFα fluorescence signals were found in kidney glomeruli from all experimental conditions. Low FKBP51 signals were observed in control, CIL and CisPt groups, while a clear FKBP51 signal was observed in glomerular cells (star) and capsule cells (red arrow) in the CisPt + CIL group. A significant decrease in the number of blebbing cells in this group versus the cisplatin-treated group is also observed (Figure 4, Figure 5 and Figure 6), at a ratio of 5.5:1 CisPt:CisPt + Cil (*p* > 0.05).

### 3.6. Expression of AmotL2 in Cisplatin-Treated and Cilastatin-Protected Rat Kidney

The cytosol of kidney tubule cells showed medium intensity, homogeneously distributed AmotL2-specific staining (bold white arrows, Control panel, Figure 5). In some cells, immunofluorescence is most prominent around nuclei (thin white arrows). Some cells of the outer glomerular capsule showed high staining intensity for AmotL2 (white arrowheads). Glomerular cells showed low AmotL2 immunostaining (star).

Tubular cells of cilastatin-treated rats (CIL panels, Figure 5) presented a homogeneous AmotL2 signal with similar localization to controls, but at a slightly but definitely increased intensity. AmotL2 staining was more intense around the nuclei of some cells, and in most nuclei appeared in a dot-like fluorescence pattern. Immunostaining for TNFα and AmotL2 was seen in the luminal side of endothelial cells of medium-sized vessels (red arrows, CIL panels, Figure 5).

AmotL2/TNFα highly positive cells were found in some tubule cells of cisplatin-treated rats (bold white arrows, CisPt panels, Figure 5). Thin white arrows point to some other cells within the same tubules which show a much lower staining intensity. In both cases, staining intensity was higher at the apical brush border than in the cytosol. AmotL2/TNFα positive cells were found in the outer glomerular capsule (white arrowheads). AmotL2 fluorescence was lower in cells with high TNFα+ staining.

Kidney tubule cells of cilastatin-protected cisplatin-treated rats showed a staining pattern very similar to controls, although with TNFα and AmotL2 at a slightly lower intensity (CisPt + CIL panels, Figure 5).

### 3.7. Expression of TNFα and IQGAP1 in Cisplatin Treated and Cilastatin Protected Rat Kidney

Low-intensity immunostaining was seen for TNFα and IQGAP1 in the cytosol of tubular cells (Control panels, Figure 6). Thin yellow arrows point to more intense IQGAP1 fluorescence around nuclei in some tubule cells. The outer glomerular capsule had small areas of cells with higher IQGAP1 immunofluorescence (white arrows). Glomerular cells showed low levels of IQGAP1-specific immunostaining.

Kidney tubule cells from cilastatin-treated rats (CIL panels, Figure 6) showed slightly higher levels of TNFα and IQGAP1 than controls, but with very similar localization. Glomerular cells showed a slightly more intense signal for IQGAP1.

The CisPt panels in Figure 6 show a clear sample of high-intensity TNFα immunofluorescence in the cytosol of some tubule cells, inside the tubular light, as pluricellular blebbing bodies (white arrows). Red arrows indicate TNFα fluorescence at the luminal-apical border of some tubular cells; however, the immunofluorescence in most of the tubules of the field is at a much lower intensity (similar to controls) in the cell cytosol (arrowheads).

Cilastatin-protected cisplatin-treated rats presented homogeneous fluorescence signals for both TNFα and IQGAP1 in kidney tubular cells. TNFα was cytoplasmic and at a similar level to controls. A few tubular cells show a TNFα-specific fluorescence signal (red arrow). IQGAP1 fluorescence is of higher intensity than in rats treated with either cilastatin or cisplatin alone. TNFα and IQGAP1 fluorescence was of very low intensity in kidney glomeruli in all experimental conditions.

Table 2 shows the intensities of specific fluorescence signals for TNFα, FKBP51, AmotL2 and IQGAP1 in different kidney cell types from the four experimental conditions.

## 4. Discussion

Previous reports from our group showed histological and functional damage and increased inflammation in rat kidneys after cisplatin administration [1]. In the current study, we confirm significant damage to renal function (Table 1), intratubular protein cast (Figure 1), and increased NF-κB (Figure 2). We also present a tubule-by-tubule and cell-by-cell analysis, which was necessary to determine the distribution of the damage.

As shown in Figure 4 and summarized in Table 2, the immunophilin FKBP51 is expressed at a basal level in the cytosol of all kinds of tubule cells and was not affected significantly by cilastatin treatment. After cisplatin treatment, total-kidney NF-κB levels increased (Figure 2); however, confocal microscopy analysis showed that the expression levels of FKBP51 and TNFα seem to be inversely related. TNFα+ tubular cells showed lower levels of FKBP51 protein, while those with basal or lower levels of TNFα expression had much higher levels of FKBP51, not only in the cytosol, but also in nuclei. The immunophilins FKBP51 and FKBP52 regulate the activity of the NF-κB family of transcription factors, which are involved in a large number of different cell functions, including cell growth and development, differentiation, inflammation, and many others. The most frequent NF-κB dimer is p50/RelA(p65). FKBP51 can have inhibitory or stimulatory effects on NF-κB signaling, depending on the type of cell [16,38,39,40].

FKBP51 seems to protect against the inflammatory effects of cisplatin, as suggested by the low expression of TNFα, a marker of inflammation. The mechanism is likely threefold. Firstly, FKBP51 plays an essential role in the TNFα/NF-κB inflammation signaling pathway [16,38,39], with recent studies showing that FKBP51 impairs nuclear translocation of the p50_RelA/p65 complex, blocking the transcriptional activity of NF-κB [40,41] and consequently preventing inflammation. Secondly, FKBP51 acts as a regulator of the glucocorticoid receptor signaling pathway, and thirdly, FKBP51 acts in a more complex way, through molecular chaperoning of metabolism, with FKBP51 independently and directly regulating phosphorylation cascades and nuclear receptors [42].

These three mechanisms of action may partly explain the pleiotropic effects of FKBP51 under cisplatin toxicity in different regions and cell types of the kidney. FKBP51 expression in kidney tubule cells of cilastatin-protected cisplatin-treated rats was higher than in controls, with no expression or non-significant expression of TNFα, and low levels of NF-κB. Nuclear FKBP51 expression in this group is even higher than in the cytosol of most tubule cells. This suggests an agonistic effect of cilastatin and cisplatin on FKBP51 expression, probably through a complex mechanism that, although outside the scope of this study, would be worthy of future investigation. The fact that cilastatin and cisplatin together led to increased FKBP51 levels suggests that early antiapoptotic action mediated by FKBP51 [22] may be at least partly responsible for this group’s lower levels of cell blebbing, which is representative of cell death by apoptosis or ferroptosis [43].

Additionally of interest for further study is FKBP51s, a C-terminus-lacking isoform which is generated by alternative splicing of FKBP5 pre-mRNA. This isoform lacks the TPR domain and can be found in nuclei [23] acting as a transcriptional regulator. Further research into the functional association of immunophilins with Hsps, especially with Hsp90, will also be of particular interest. The alternative supramolecular heterocomplexes formed (NFκB, FKBP51, FKBP52, Hsps, hTERT, glucocorticoid receptor, etc.) are critical to the many pleiotropic effects previously mentioned, ultimately deciding the fate of the cell. Further study in this area is particularly warranted given that the effects vary in different types of cells [14,16,40].

Although cisplatin did not affect AmotL2 expression in kidney tubule cells with basal levels of TNFα (Figure 5 and Table 2), in inflamed TNFα+ cells, AmotL2 intensity levels were higher than in surrounding TNFα± cells. The addition of cilastatin seems to reduce TNFα expression and homogenize AmotL2 expression to control levels. AmotL2 localization seems to be always cytosolic, usually of homogeneous distribution, but more intense in the nuclei of blebbing cells, either in samples from control or cilastatin-treated rats (Figure 5) but apparently never in cisplatin-treated or cilastatin-protected cisplatin-treated rats. The increased expression of AmotL2 by cilastatin returning to basal levels under cisplatin co-administration may indicate a pre-stress activation that prevents cisplatin-induced inflammation (TNFα−).

No changes in intensity or localization were detected for AmotL2 in Bowman´s external capsule cells, or glomerular or vessel cells.

The absence of changes in AmotL2 expression levels either in vessels or glomerular capillaries under either combined or single treatment with cilastatin or cisplatin suggests that the role of AmotL2 in this process is as a scaffold protein regulating pools of transcription factors like LATS1/2 (Large Tumor Suppressor Kinase) [44], which ultimately regulates the key downstream target YAP (yes-associated protein) [24] in the Hippo signaling pathway. Further investigation is required to resolve this issue, especially in the light of conflicting research publications, some of which have identified angiomotin proteins as potent suppressors of YAP, while others have shown angiomotins to be YAP activators [15,44,45]; furthermore, the few studies which have looked at YAP-TNFα interrelationships have also yielded conflicting results. For example, reciprocal stimulation of TNFα and YAP signaling activities has been reported in renal tubules [46], while in MC3T3-E1 cells, YAP1 expression was downregulated after treatment with TNFα, and YAP1 attenuated the TNFα-induced activation of the NF-κB signaling pathway [47].

Cilastatin increases IQGAP1 expression along with TNFα in renal tubule cells, both in a homogeneous manner (Table 2 and Figure 6). Cisplatin elicits a higher increase of IQGAP1 only in TNFα− cells; however, cilastatin and cisplatin together increase the intensity in both TNFα− and + cells, although to a higher level in TNFα− cells (Table 2 and Figure 6). No nuclear localization was seen in any kind of cell. Both combined and individual treatment with cilastatin or cisplatin led to a slight increase in IQGAP1 expression in glomerular and glomerular capsule cells but did not lead to compartmentalized subcellular localization.

Aptly described as a “molecular puppeteer”, IQGAP1 is a scaffold of, among others, the core proteins of the Hippo pathway [48], facilitating crosstalk between the Hippo network and the AKT and ERK pathways, and negatively regulating the pro-apoptotic signal mediated by this pathway [48,49,50]. This is consistent with our results, and it is conceivable that IQGAP1 and AmotL2 play complementary scaffolding roles in the Hippo-YAP pathways involved in tubule cell escape from cisplatin-induced apoptosis; however, the interaction between YAP and TNFα/NF-κB pathways seems to depend on cell type and context, as evidenced by their opposite effects in chondrocytes [51] and LPS-induced endothelial inflammation [52].

Anoikis is a form of anchorage-dependent cell death caused by the loss of cell matrix and consecutive detachment of cells (blebbing), a form of tubular cell death due to heavy metal toxicity [53]. IQGAP1, interacting with cytoskeletal proteins [25,26], may be an active component in cilastatin-mediated protection against cell death [54], thus preventing cisplatin toxicity.

Finally, recently it has been shown that miR-124, a 3′-UTR of IQGAP1, might be associated with the development of inflammation in liver fibrosis [55]. Overexpression of miR-124 and knockdown of IQGAP1 led to downregulation of TNFα, IL-1β and IL-6, while knockdown of miR-124 or overexpression of IQGAP1 produced the opposite result. When compared with the findings of the present study, these results point to the activation of different pathways in different cell types, or by different causes of inflammation.

Variations described in capsule and glomerular cells do not appear to be related to inflammation and are outside the scope of this article, but would nonetheless be of interest for further study.

Our findings offer significant insights into the involvement of FKBP51, AmotL2 and IQGAP1 in kidney tubule cisplatin toxicity and its prevention by cilastatin. The precise roles of these three proteins and their functional co-involvement in these processes pose interesting new questions for future research.

## 5. Conclusions

The renal inflammation caused by cisplatin toxicity is focally and segmentally localized, in some tubules grouped in foci, and to different degrees in different cells within the same tubule. Cilastatin greatly reduces the levels of inflammation (although not to basal level) and slightly increases FKBP51, AmotL2 and IQGAP1 protein expression.

Cisplatin treatment modifies renal tubule FKBP51, AmotL2 and IQGAP1 expression in a cellular- and subcellular-specific manner. Expression is also dependent on the level of inflammation in the tubule cells. Cells expressing high levels of FKBP51 have no or very low expression of TNFα. Conversely, cells expressing TNFα have low levels of FKBP51. AmotL2 and TNFα seem to colocalize and their expression is increased in tubular cells. There is a complex relationship between changes in IQGAP1 expression/localization and TNFα. Finally, treatment with cilastatin and cisplatin together leads to a slight increase in IQGAP1 expression in glomerular and glomerular capsule cells.

## Figures and Tables

**Table 1:** Effects of cilastatin treatment on cisplatin-induced renal toxicity in rats. Results are expressed as mean ± SEM. ^a^ *p* < 0.001, ^b^ *p* < 0.005 vs. control and control + cil; ^c^ *p* < 0.05 vs. control + cil; ^d^ *p* < 0.0001, ^e^ *p* < 0.001, ^f^ *p* < 0.0005 vs. all other groups. Cil: cilastatin; S_Creat_: serum creatinine; BUN: blood urea nitrogen; GFR: glomerular filtration rate; U_Vol_: urinary volume; FE: fractional excretion.

Groups	S_Creat_ (mg/dL)	BUN (mg/dL)	GFR (mL/min/100 g)	U_Vol_ (mL/24 h)	FE_Na_^+^ (%)	FE_H2O_ (%)
Control	0.29 ± 0.02	27.14 ± 1.65	0.76 ± 0.05	16.00 ± 3.49	0.46 ± 0.04	0.53 ± 0.13
Control + Cil	0.29 ± 0.02	26.29 ± 2.36	0.72 ± 0.05	14.21 ± 2.05	0.47 ± 0.06	0.48 ± 0.05
Cisplatin	1.48 ± 0.12 ^d^	108.57 ± 12.67 ^d^	0.14 ± 0.02 ^e^	29.00 ± 3.12 ^b^	1.55 ± 0.30 ^f^	5.55 ± 0.84 ^d^
Cisplatin + Cil	0.63 ± 0.11 ^a^	50.71 ± 12.48	0.46 ± 0.09 ^b^	23.29 ± 2.06 ^c^	0.64 ± 0.13	1.49 ± 0.25

**Table 2:** Intensity of specific fluorescence signal for TNFα, FKBP51, AmotL2 and IQGAP1 in kidney cell types from control, cilastatin-treated (CIL), cisplatin-treated (CisPt), cilastatin-protected cisplatin-treated (CisPt + CIL) groups of rats. Intensity levels: ± basal-faint, + low, ++ medium, and +++ high.

**TNFα**		**Control**	**CIL**	**CisPt**	**CisPt + CIL**
**Tubule cells**		±	±	±/+++	+/++
**Ext. capsule cells**		±/++	±/++	±/++	±/++
**Glomerulus cells**		±	±	±	±
**FKBP51**		**Control**	**CIL**	**CisPt**	**CisPt + CIL**
**Tub. cells**	TNFα±	basal	±	±	++/+++
	TNFα+	±/++	±/++	+++	+/+++
**Ext. capsule cells**		±	±	±	++
**Glomerulus cells**		±	±	±	++
**AmotL2**		**Control**	**CIL**	**CisPt**	**CisPt + CIL**
**Tub. cells**	TNFα±	basal	±	±	±/++
	TNFα+	++	++/+++	+++	±/++
**Ext. capsule cells**		±/++	±/++	±/++	±/++
**Glomerulus cells**		±/++	±/++	±/++	±/++
**IQGAP1**		**Control**	**CIL**	**CisPt**	**CisPt + CIL**
**Tub. cells**	TNFα±	basal	±	±	+++
	TNFα+	±	±/++	±/++	++
**Ext. capsule cells**		±	±/++	±/++	±/++
**Glomerulus cells**		±	+	+	+

## Data Availability

Not applicable.

## Dedicatory

Authors dedicate this article to the memory of Professor Alberto Tejedor, teacher, colleague and friend, who started the study of cilastatin as a kidney protector.
